# A survey of national ethics and bioethics committees 

**DOI:** 10.2471/BLT.19.243907

**Published:** 2020-11-30

**Authors:** Johannes Köhler, Andreas Alois Reis, Abha Saxena

**Affiliations:** a Department of Anesthesiology and Critical Care Medicine, Kantonsspital Münsterlingen, Spitalcampus 1, 8596 Münsterlingen, Switzerland.; b Health Ethics and Governance Unit, World Health Organization, Geneva, Switzerland.; c Institut Éthique Humanité Histoire, University of Geneva, Geneva, Switzerland.

## Abstract

**Objective:**

To assess the current state of national ethics committees and the challenges they face.

**Methods:**

We surveyed national ethics committees between 30 January and 21 February 2018.

**Findings:**

In total, representatives of 87 of 146 national ethics committees (59.6%) participated. The 84 countries covered were in all World Bank income categories and all World Health Organization regions. Many national ethics committees lack resources and face challenges in several domains, like independence, funding or efficacy. Only 40.2% (35/87) of committees expressed no concerns about independence. Almost a quarter (21/87) of committees did not make any ethics recommendations to their governments in 2017, and the median number of reports, opinions or recommendations issued was only two per committee Seventy-two (82.7%) national ethics committees included a philosopher or a bioethicist.

**Conclusion:**

National ethics (or bioethics) committees provide recommendations and guidance to governments and the public, thereby ensuring that public policies are informed by ethical concerns. Although the task is seemingly straightforward, implementation reveals numerous difficulties. Particularly in times of great uncertainty, such as during the current coronavirus disease 2019 pandemic, governments would be well advised to base their actions not only on technical considerations but also on the ethical guidance provided by a national ethics committee. We found that, if the advice of national ethics committees is to matter, they must be legally mandated, independent, diverse in membership, transparent and sufficiently funded to be effective and visible.

## Résumé

**Objectif:**

Évaluer l'état actuel des comités nationaux d'éthique et les difficultés qu'ils rencontrent.

**Méthodes:**

Nous avons mené l'enquête auprès des comités nationaux d'éthique entre le 30 janvier et le 21 février 2018.

**Résultats:**

Au total, les représentants de 87 des 146 comités nationaux d'éthique (59,6%) ont participé. Les 84 pays retenus étaient issus de toutes les catégories de revenus définies par la Banque mondiale, ainsi que de toutes les régions délimitées par l'Organisation mondiale de la Santé. Nombre de ces comités manquent de ressources et sont confrontés à des problèmes dans divers domaines tels que l'indépendance, le financement ou l'efficacité. Seulement 40,2% (35/87) d'entre eux n'ont exprimé aucune inquiétude quant à leur indépendance. Presque un quart (21/87) des sondés n'ont adressé aucune recommandation éthique à leur gouvernement en 2017, et le nombre médian de rapports, d'opinions ou de recommandations publiés ne dépassait pas deux par comité. Pas moins de 72 (82,7%) comités nationaux d'éthique incluaient dans leurs rangs un philosophe ou un bioéthicien.

**Conclusion:**

Les comités nationaux d'éthique (ou de bioéthique) émettent des recommandations et orientations pour les gouvernements et les citoyens, afin que les politiques publiques tiennent compte des enjeux éthiques. Bien que la tâche paraisse simple, la mise en œuvre s'avère nettement plus compliquée. Au moment d'agir, les gouvernements ont tout intérêt à s'inspirer non seulement des considérations techniques, mais aussi des conseils fournis par un comité national d'éthique. A fortiori lorsque l'époque est marquée par une grande incertitude, comme aujourd'hui avec la pandémie de maladie à coronavirus 2019. Force est de constater que, pour que leurs avis pèsent dans la balance, les comités nationaux d'éthique doivent disposer d'un mandat légal, être indépendants et transparents, afficher une grande diversité de membres, et être dotés d'un budget suffisant pour garantir leur efficacité et leur visibilité.

## Resumen

**Objetivo:**

Evaluar el estado actual de los comités nacionales de ética y los desafíos que conllevan.

**Métodos:**

Realizamos una encuesta a los comités nacionales de ética entre el 30 de enero y el 21 de febrero de 2018.

**Resultados:**

En total, participaron representantes de 87 de los 146 comités nacionales de ética (59,6%). Los 84 países seleccionados procedían de todas las categorías de ingresos definidas por el Banco Mundial, así como de todas las regiones definidas por la Organización Mundial de la Salud. Muchos de esos comités carecen de recursos suficientes y se enfrentan a problemas en diversas esferas, como la independencia, la financiación o la eficacia. Solo el 40,2% (35/87) de ellos no expresó ninguna preocupación por su independencia. Casi una cuarta parte (21/87) de los encuestados no hizo ninguna recomendación ética a su gobierno en 2017, y el número medio de informes, opiniones o recomendaciones publicadas no superó los dos por comité. Hasta 72 (82,7%) Comités Nacionales de Ética incluyeron a un filósofo o bioeticista entre sus miembros.

**Conclusión:**

Los comités nacionales de ética (o bioética) proporcionan recomendaciones y orientación a los gobiernos y a los ciudadanos, a fin de que las políticas públicas tengan en cuenta las cuestiones éticas. Aunque la tarea parece sencilla, su aplicación revela numerosas dificultades. Especialmente en tiempos de gran incertidumbre, como durante la actual pandemia de la enfermedad coronavirus 2019, los gobiernos deberían basar sus acciones no solo en consideraciones técnicas sino también en la orientación ética proporcionada por un comité nacional de ética. Hemos comprobado que, para que el asesoramiento de los comités nacionales de ética sea importante, estos deben tener un mandato legal, ser independientes, tener una composición diversa, ser transparentes y estar suficientemente financiados para ser eficaces y visibles.

## ملخص

الغرض تقييم الوضع الحالي للجان الأخلاقيات الوطنية، والتحديات التي تواجهها.

الطريقة قمنا بمسح لجان الأخلاقيات الوطنية ما بين 30 يناير/كانون ثاني و21 فبراير/شباط من عام 2018.

النتائج بشكل إجمالي، شارك ممثلون عن 87 لجنة من 146 لجنة وطنية للأخلاقيات (59.6%). كانت الدول المشمولة البالغ عددها 84 دولة، تمثل جميعها كل فئات الدخل التابعة للبنك الدولي، وكل المناطق التابعة لمنظمة الصحة العالمية. تفتقر العديد من لجان الأخلاقيات الوطنية إلى الموارد، وتواجه تحديات في العديد من القطاعات، مثل الاستقلال، أو التمويل، أو الفعالية. فقط 40.2% (35/87) من اللجان لم تعرب عن مخاوف بخصوص الاستقلال. لم تقدم ربع اللجان تقريباً (21/87) أية توصيات أخلاقية إلى حكوماتها في عام 2017، وكان متوسط ​​عدد التقارير، أو الآراء، أو التوصيات الصادرة هو اثنين (2) فقط لكل لجنة. اشتملت عضوية اثنتان وسبعون لجنة أخلاقيات وطنية (82.7%) على فيلسوف أو عالم أخلاقيات بيولوجية.

الاستنتاج تقدم لجان الأخلاقيات الوطنية (أو الأخلاقيات الحيوية) توصيات وتوجيهات للحكومات والشعوب، وبالتالي ضمان أن السياسات العامة على دراية بالمخاوف الأخلاقية. وبالرغم من أن هذه المهمة تبدو مباشرة، إلا أن التنفيذ يكشف عن العديد من الصعوبات. وفي أوقات عدم اليقين الشديد على وجه الخصوص، مثل وباء فيروس مرض فيروس كورونا الحالي 2019، تُنصح الحكومات عن حق بأن تبني إجراءاتها ليس فقط على الاعتبارات الفنية، ولكن أيضًا على التوجيهات الأخلاقية التي تقدمها لجنة الأخلاقيات الوطنية. لقد اكتشفنا أنه إذا كانت مشورة لجان الأخلاقيات الوطنية ذات جدوى، فإنه يجب أن تكون مفوضة بشكل قانوني، ومستقلة، ومتنوعة في العضوية، وتتمتع بالشفافية، وتحصل على تمويل كاف، لتكون فعالة وذات أثر واضح. ****

## 摘要

**目的:**

旨在评估国家伦理委员会的现状及其面临的挑战。

**方法:**

我们在 2018 年 1 月 30 日至 2 月 21 日期间对国家伦理委员会进行了调查。

**结果:**

总共 146 个国家伦理委员会中的 87 个代表 (59.6%) 参加了这项调查。涉及的 84 个国家分布于世界银行的所有收入类别和世界卫生组织的所有区域。许多国家伦理委员会缺乏资源并且在独立性、资金或效率等方面面临挑战。仅 40.2% (35/87) 的委员会表示不担心独立性问题。在 2017 年，近四分之一 (21/87) 的委员会并未向其政府提出任何伦理建议，发布的报告、意见或建议的中位数仅为每个委员会两份。七十二个 (82.7%) 国家伦理委员会拥有一位哲学家或一位生物伦理学家。

**结论:**

国家伦理（或生物伦理）委员会向政府和公众提供建议和指导，从而确保公共政策以伦理问题为依据。尽管该任务看似简单，但实施过程却暴露出许多困难。尤其是在充满不确定性的时候，例如在当前持续的新型冠状病毒肺炎大流行期间，建议各国政府不仅需要根据技术因素，而且还应根据国家伦理委员会提供的伦理指导采取行动。我们发现，如果希望国家伦理委员会的建议得到重视，则这些委员会必须获得法律授权、具有独立性、成员多样性、透明性并得到充足的有效可用资金支持。****

## Резюме

**Цель:**

Оценить текущее состояние национальных комитетов по этике и проблемы, с которыми они сталкиваются.

**Методы:**

Исследование среди национальных комитетов по этике проводилось в период с 30 января по 21 февраля 2018 г.

**Результаты:**

Всего 87 из 146 национальных комитетов по этике (59,6%) приняли участие в исследовании. Участвовавшие в исследовании 84 страны представляли все категории доходов по оценке Всемирного банка и все регионы Всемирной организации здравоохранения. Многие национальные комитеты по этике испытывают нехватку ресурсов и проблемы в ряде таких сфер, как независимость, финансирование или эффективность работы. Только 40,2% (35 из 87) комитетов заявили, что они не испытывают проблем с точки зрения своей независимости. Почти четверть (21 из 87) комитетов не давали этических рекомендаций правительствам в 2017 году, а среднее количество отчетов, мнений или рекомендаций, которые выпустил каждый комитет, составило всего две единицы. В семидесяти двух национальных комитетах по этике (82,7%) в состав входил специалист по философии или биоэтике.

**Вывод:**

Национальные комитеты по этике (или биоэтике) направляют рекомендации и дают наставления правительствам и общественности, благодаря чему этические проблемы находят отражение в общественной политике. Хотя эта задача представляется весьма простой, ее осуществление связано с многочисленными сложностями. В частности, в периоды большой неопределенности, например во время текущей пандемии коронавирусной инфекции 2019 года, правительствам было бы целесообразно действовать не только с учетом технических соображений, но и с учетом этических рекомендаций национального комитета по этике. Авторы обнаружили, что если предполагается прислушиваться к советам национальных комитетов по этике следует, то они для этого должны обладать законодательно закрепленными полномочиями и должны быть независимыми, разнообразными по представительству, прозрачными и в достаточной мере финансируемыми. Тогда их деятельность будет заметной и эффективной.

## Introduction

Emerging technologies such as gene editing and artificial intelligence touch upon the fundamental question of what it means to be human. The innovative use of data and social media to improve public health questions the very notions of privacy and confidentiality. And global events, such as climate change, environmental disasters and disease outbreaks, raise concerns about access to resources and their equitable use. The ethical consequences of these developments can lead to social and societal tensions – especially in an unequal world undergoing rapid demographic change. Crafting effective policies, laws and regulations in response to these developments, while considering their varied social, cultural, political and historical contexts and the ethical dilemmas raised, is far from easy. In pluralistic societies, public opinion must be taken into consideration. Consequently, governments need robust mechanisms for managing bioethical issues that reflect the diverse opinions not only of scientists but also of physicians, lawyers, philosophers, laypeople, communities and other individuals who can advise policy-makers and governments on the best course of action. Together, they can identify and clarify the values at stake and the ethical principles that must be upheld. To be credible, such mechanisms must ensure that divergent views are acknowledged and that a consensus is achieved in a systematic fashion that involves transparent ethical reasoning in accordance with pre-established rules. Intuitive as this idea sounds, such mechanisms were not implemented systematically until relatively recently, partly because scientists and physicians had a professional resistance to external scrutiny.^1^^–^^3^

The principal mechanism adopted by countries to tackle these issues is the national ethics committee, also termed the national ethics commission or national bioethics committee or commission. The United States of America (USA) established such a committee through the 1974 National Research Act, one of whose functions was to “undertake a comprehensive study of the ethical, social, and legal implications of advances in biomedical and behavioral research and technology”.^4^ Elsewhere, progress was slow. In Europe, France was first to establish a national ethics committee in 1983. Since 1992, committees in Europe have been facilitated by the European Conference of National Ethics Committees, which is sponsored by the Council of Europe.^5^ In most European countries, committees were eventually inscribed into law. The form and level of activity of these committees depend on the sociopolitical context in which they operate – most are advisory. National ethics committees are usually mandated through a juridical process, they contribute to discussions in both the public domain and parliament, and information about their function and activities is widely available. In low- and middle-income countries where no comparable regional catalyser existed, it was a 2005 declaration by the United Nations Educational, Scientific and Cultural Organization (UNESCO) that urged countries to develop independent, multidisciplinary and pluralistic national ethics committees (Box 1).^6^ Today, UNESCO provides support through the Assisting Bioethics Committees programme by working with ministries and government to establish committees in countries requesting assistance.

Box 1 **UNESCO universal declaration on bioethics and human rights^6^**

Article 19 – Ethics committees

Independent, multidisciplinary and pluralist ethics committees should be established, promoted and supported at the appropriate level to:

(i) assess the relevant ethical, legal, scientific and social issues related to research projects involving human beings;

(ii) provide advice on ethical problems in clinical settings;

(iii) assess scientific and technological developments, formulate recommendations and contribute to the preparation of guidelines on issues within the scope of this declaration; and

(iv) foster debate, education and public awareness of, and engagement in, bioethics.

UNESCO: United Nations Educational, Scientific and Cultural Organization.

Establishing national ethics committees is merely the first step – the greater challenge is to increase their capacity to be independent, pluralistic, enquiring bodies able to give sustainable advice to governments and the public. As Gefenas & Lukaseviciene point out,^5^ “It is important to ensure that the new institutions are not established merely to comply formally with the recommendation to create a national committee but, rather, that they satisfy genuine needs to deal with urgent and country-specific bioethical concerns”. The struggles of national ethics committees, particularly in low- and middle-income countries, have been debated and discussed at biennial Global Summits of National Bioethics Committees. These Global Summits provide an opportunity for representatives of national ethics committees to share information and experiences and to deliberate on a wide range of prominent ethical topics. Since 2004, the World Health Organization (WHO) has provided a permanent secretariat for these events. In that time, the team at WHO has been involved in supporting national ethics committees to host these events and in working with representatives of committees from different countries, including countries looking to establish a national ethics committee. This experience has given the team a unique insight into the issues and challenges facing these committees.

Although some literature is available on national ethics committees in specific countries or regions and some globally relevant background information exists,^7^^–^^14^ there is a lack of up-to-date empirical data on,^13^ for example, the needs and strengths of national ethics committees internationally, particularly those in low- and middle-income countries.^5^ Given the vital role these committees play in providing independent, well founded ethical advice to government and the public on key issues, it is crucial we understand the challenges they face and the conditions under which these committees can thrive. Consequently, we explored the current state of national ethics committees worldwide by carrying out a survey of committee representatives. Based on the findings of that survey, we discuss here the key challenges national ethics committees face today and how, in our opinion, their sustained success can be ensured.

## Methods

A research protocol was drafted and modified following comments from two independent reviewers. We carried out a cross-sectional survey, which was administered in English through WHO’s version of LimeSurvey (LimeSurvey GmbH, Hamburg, Germany), a free, open-source online-survey application. Data were collected between 30 January and 21 February 2018. We contacted representatives of 146 national ethics committees by email and invited them to take part. The analysis was conducted using LimeSurvey’s built-in statistical functions and Microsoft Excel 2010 with the Analysis ToolPak (Microsoft Corporation, Redmond, USA). Given the sensitivity of the information obtained, we refrained from publishing data that could identify individual national ethics committees. All participants provided informed consent and the study was exempted from review by WHO’s research ethics review committee.

## Results

**Fig. 1:**
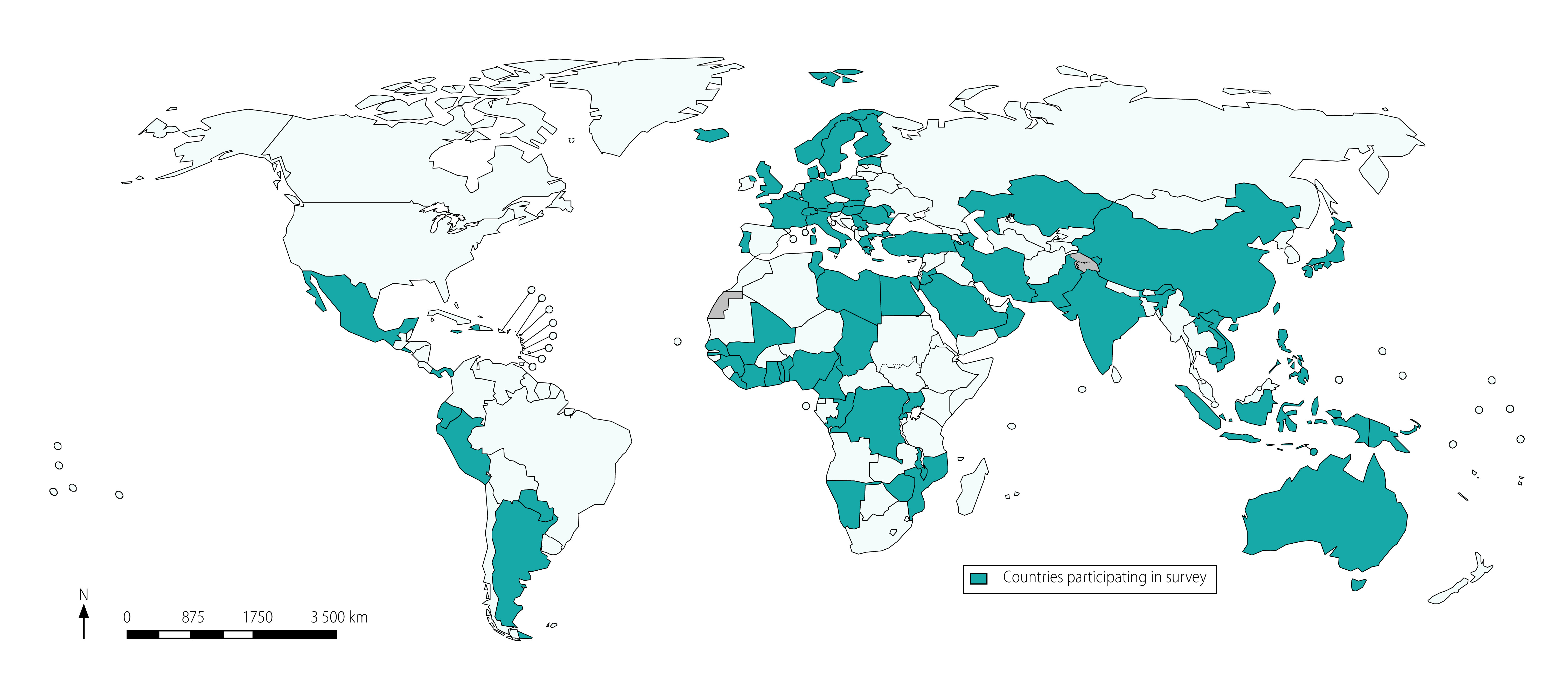
Participating countries, survey of national ethics committees, 2018

Note: Included countries are Argentina, Australia, Austria, Azerbaijan, Bangladesh, Belgium, Benin, Cambodia, Cameroon, Chad, China, Congo, Côte d'Ivoire, Cyprus, Democratic Republic of the Congo, Denmark, Ecuador, Egypt, El Salvador, Estonia, Fiji, Finland, France, Gambia, Germany, Ghana, Greece, Guinea, Haiti, Hungary, Iceland, India, Indonesia, Iran (Islamic Republic of), Italy, Jamaica, Japan, Jordan, Kazakhstan, Kyrgyzstan, Lao People's Democratic Republic, Lebanon, Liberia, Libya, Luxembourg, Malawi, Mali, Malta, Mexico, Mozambique, Namibia, Nepal, Nigeria, Norway, Oman, Pakistan, Panama, Papua New Guinea, Paraguay, Peru, Philippines, Poland, Portugal, Qatar, Republic of Moldova, Romania, Rwanda, San Marino, Saudi Arabia, Senegal, Serbia, Singapore, Slovakia, Solomon Islands, Sri Lanka, Sweden, Switzerland, Togo, Tunisia, Turkey, Uganda, United Kingdom of Great Britain and Northern Ireland, Viet Nam and Zimbabwe.

Representatives of 87 of 146 national ethics committees (59.6%), who came from 84 countries, agreed to participate in the survey (Fig. 1). Countries were classified according to their World Bank income group:^15^ participants came from 16 of 34 low-income countries, 22 of 47 lower-middle-income countries, 19 of 56 upper-middle-income countries and 27 of 81 high-income countries. 

**Fig. 2:**
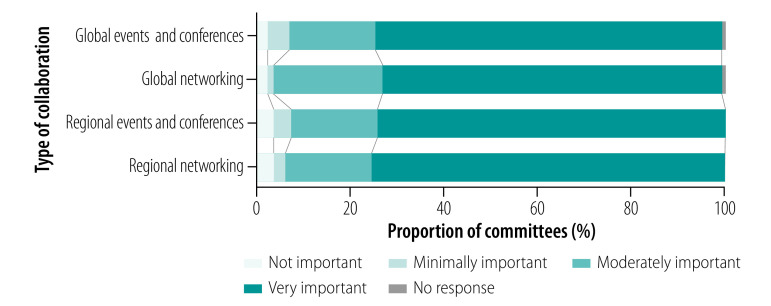
Perceived importance of global and regional collaboration, survey of national ethics committees, 84 countries, 2018

Note: Full survey findings of the 87 committees are available from the online data repository.^16^

**Fig. 3:**
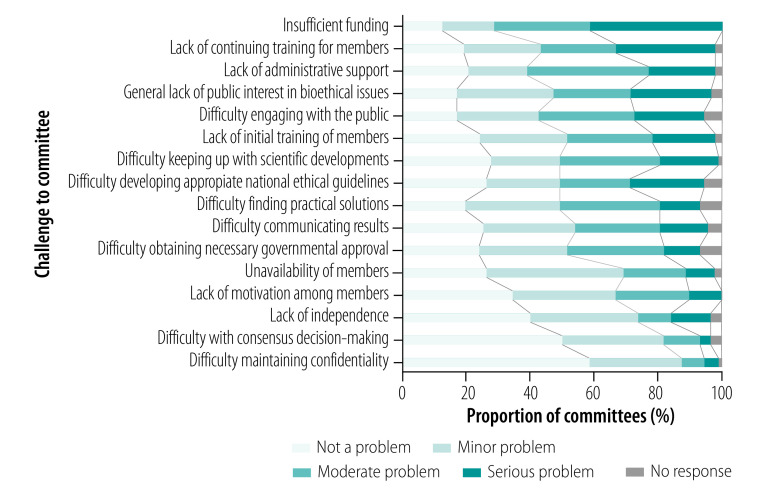
Challenges to committees, survey of national ethics committees, 84 countries, 2018

Notes: We surveyed 87 committees. Representatives of national ethics committees rated challenges to their committee using a 4-point Likert scale.

A summary of the survey’s findings are presented in Table 1, Table 2 and Fig. 2. The full findings are available from the online data repository.^16^ In particular, national ethics committees were asked to rate the relevance of various challenges to their committee on a 4-point Likert scale (Fig. 3).

**Table 1:** Selected results, survey of 87 national ethics committees, 84 countries, 2018

Parameter^a^	Value
**General (*n* = 87)**	
Affiliation, no. of committees (%)	
Ministry of health	48 (55.2)
Government	25 (28.7)
Ministry of education	10 (11.5)
UNESCO	10 (11.5)
Establishment, no. of committees (%)	
Legal act	46 (52.9)
Government	41 (47.1)
Links to government, no. of committees (%)	
Appointment of members	61 (70.1)
Funding	56 (64.4)
Premises	46 (52.9)
Appointment of chair	41 (47.1)
Under direct governmental oversight	15 (17.2)
**Membership (*n* = 87)**	
Members, median (IQR)	15 (12–20)
Female members, median (IQR)	6 (4–8.25)
Members’ background, no. of committees (%)	
Medicine	85 (97.7)
Ethics or philosophy (academia)	72 (82.8)
Law	67 (77.0)
Other social sciences	63 (72.4)
**Activity (*n* = 87)**	
Meetings each year, median (IQR)	8 (3.75–12)
Publications of opinions, reports, policy recommendations or a combination (no.),^b^ median (IQR)	2 (0.5–5.5)
Involvement in preparation of work and decisions, no. of committees (%)	
Receipt of expert technical advice	77 (88.5)
Literature reviews	72 (82.8)
Stakeholder hearings	58 (66.7)
Public consultations	29 (33.3)
Decision-making method, no. of committees (%)	
Consensus only	45 (51.7)
Voting only	9 (10.3)
Both or mixed	31 (35.6)
Contributions to:	
health-related laws and regulations, median (IQR)	1 (0–2)
health-related policies, median (IQR)	1 (0–3)
**Funding**	
Proportion of funding coming from government,^c^ median (range) (*n* = 82)	90 (0–100)
Number of sources, no. of committees (%) (*n* = 74)	
> 1	30 (40.5)
> 2	8 (10.8)
**Secretariat**	
Secretariat available, no. of committees (%) (*n* = 87)	79 (90.8)
Funding of secretariat, no. of committees (%) (*n* = 79)	
Directly by government	42 (53.2)
Through committee’s operational budget	24 (30.4)
Review fee	3 (3.8)
Parliament	2 (2.5)
Tasks, no. of committees (%) (*n* = 79)	
Organizing meetings	72 (91.1)
Preparing responses	60 (75.9)
Preparing written opinions, reports and policy recommendations	56 (70.9)
Staff (no.), median (IQR)	3 (2–5)
Full-time equivalent employees	
Available, median (IQR)	2 (1–3.5)
Needed, median (IQR)	4 (2–5.5)
Ratio of staff available to staff needed,^d^ average	0.65
Committees whose needs were met (i.e. ratio: ≥ 1), no. (%) (*n* = 77)	12 (15.6)
Compensation of staff, no. of committees (%) (*n* = 79)	
Fixed salary	50 (63.3)
Attendance fee	13 (16.5)
Reimbursement of travel costs	22 (27.8)
No financial compensation	15 (19.0)
**Public relations and engagement (*n* = 87)**	
Annual press releases,^e^ median (IQR)	0 (0–2)
Cooperation with broadcast media (no. occasions),^f^ median (IQR)	1.5 (0–3.25)
No press releases and no cooperation with broadcast media, no. of committees (%)	31 (35.6)
Estimated proportion of bioethics topics involving the committee in 2017 that were picked up by the country’s media, no. of committees (%)	
75–100%	6 (6.9)
50–74%	6 (6.9)
24–49%	12 (13.8)
0–24%	45 (51.7)
No estimate	18 (20.7)
Website used, no. of committees (%)	
Own	47 (54.0)
Government	30 (34.5)
Social media use, no. of committees (%)	
Facebook	8 (9.2)
Twitter	4 (4.6)
LinkedIn	1 (1.1)

IQR: interquartile range; UNESCO: United Nations Educational, Scientific and Cultural Organization.

^a^ Full survey findings are available from the online data repository.^16^

^b^ Overall, 24.1% (21/87) of committees published no opinions, reports or policy recommendations in 2017.

^c^ The average proportion of funding that came from government was 58.9%.

^d^ The standard deviation was ± 0.79 and the range was 0–5.

^e^ Overall, 55.2% (48/87) of committees issued no press releases in 2017 and 12.6% (11/87) issued more than four.

^f^ Overall, 39.1% (34/87) of committees cooperated with broadcast media on no occasions, whereas 23.0% (20/87) cooperated on more than four occasions

**Table 2:** Funding sources of 74 national ethics committees, 72 countries, 2018

Funding source	No. of respondents (%)				
	Ranking of source (*n* = 87)				Mentioned source^a,b^ (*n* = 74)
	First	Second	Third		
Government	45 (51.7)	8 (9.2)	2 (2.3)		55 (74.3)
Fees for protocol reviews	15 (17.2)	10 (11.5)	0 (0.0)		26 (35.1)
Parliament	5 (5.7)	0 (0.0)	1 (1.1)		6 (8.1)
International organizations	3 (3.4)	2 (2.3)	1 (1.1)		8 (10.8)
Other	3 (3.4)	2 (2.3)	2 (2.3)		8 (10.8)
Public institutions	2 (2.3)	4 (4.6)	1 (1.1)		10 (13.5)
Charitable foundations	1 (1.1)	1 (1.1)	0 (0.0)		3 (4.1)
Industry	0 (0.0)	0 (0.0)	0 (0.0)		1 (1.4)
Private donors	0 (0.0)	3 (3.4)	1 (1.1)		5 (6.8)
No response^c^	13 (14.9)	57 (65.5)	79 (90.8)		NA

NA: not applicable.

^a^ Only 85.1% (74/87) of respondents revealed their committees’ financial sources.

^b^ Respondents were asked to rank up to nine funding sources, hence number of times the sources are mentioned can be higher than the sum of the ranks one to three.

^c^ No response for the first rank means respondents did not want to reveal their financial sources. No response for the second and third ranks means that these committees did not have more than one or two sources of funding.

Our survey provides data on national ethics committees across all World Bank income categories and all WHO regions. However, we cannot guarantee that we contacted every existing national ethics committee. In addition, the self-reporting nature of the survey is a limitation.

## Discussion

Current advances in science and technology, such as big data, artificial intelligence and gene editing, share some common characteristics: they are highly complex, tend to demand a global perspective (and response), and develop rapidly. Although many classical ethical issues, for example euthanasia and abortion, can be dealt with nationally within a clear jurisdiction (although “medical tourism” does occur), a range of current issues tend – by their very nature – not to respect national boundaries. Data are harvested and exploited across countries and genetically engineered plants and animals cross frontiers just as easily. Similarly, global events, such as pandemics and major disasters, raise complex issues of cross-border solidarity juxtaposed with the protection of national interests, the sharing of data, samples and resources, travel and trade restrictions, and possible stigmatization of entire countries. Where they exist, national ethics committees seem to be the obvious choice to meet these challenge.

### Role and function of national ethics committees

Although the role and function of national ethics committees vary between countries, it is generally agreed that committees must operate under a national mandate and systematically provide guidance and advice to policy-makers, governments and the public on the ethical dimensions of: (i) advances in health sciences; (ii) advances in life sciences; and (iii) innovative health policies.^17^ Responses to our survey and discussions with the chairs of national ethics committees corroborate this view. The committees’ other roles (e.g. at a global level) follow from this primary responsibility. In many low- and middle-income countries, limited bioethics capacity has often resulted in the mandate of existing national research ethics committees being expanded to include reviewing and advising on bioethical issues. Conversely, our survey reveals that some established and recognized national ethics committees may perform only the functions of a national research ethics committee.

There is a consensus that national ethics committees must be multidisciplinary to ensure a multitude of views and opinions is taken on board, including those of laypersons. However, the role of bioethicists in these committees can be contentious. Bioethics expertise on committees has been criticized from the viewpoint that it may serve hidden interests or rely on dogmatic ideologies. Moreover, there are epistemological concerns.^13^ Many critics maintain that bioethicists represent the interests of scientists and industry;^18^ others question whether expertise in ethical or moral matters even exists and, if so, what implications such expertise has for ethics committees.^19^^–^^23^ In our opinion, it might be helpful to have at least one bioethicist on the committee who can challenge, explain and justify arguments, positions and decisions and help with reasoning. Our survey revealed that 82.7% (72/87) of national ethics committees included a philosopher or a bioethicist. Gender imbalance is also pertinent: there is scope for committees to improve the representation of women, which would ensure more gender-balanced discussions, while also supporting progress towards the United Nations’ sustainable development goals.^24^ The underrepresentation of women has been argued to be particularly troubling as modern bioethics disproportionately concerns women’s bodies.^25^

Although national ethics committees provide a widespread means of institutionalizing ethics, it does not follow that governments of countries without such a committee do not incorporate ethics into their policies. Many governments establish ad hoc committees or commissions to provide guidance on specific policy issues or include ethics in health technology assessments.^26^^,^^27^ However, some commentators claim that the latter approach has not been very successful.^28^ Whether or not a national ethics committee is the right model for all countries is a difficult question. Still, assuming that such a mechanism is better at fostering consistency, transparency and accountability than ad hoc committees or commissions seems reasonable. Moreover, international collaborations and networking function better between institutions with similar mandates. And, unlike ad hoc mechanisms, national ethics committees allow for constant learning and development.

### COVID-19: a case in point

The current coronavirus disease 2019 (COVID-19) pandemic starkly demonstrates why national ethics committees are needed. The pandemic raises numerous complex ethical issues, ranging from governance of the pandemic response, the challenge of public and community engagement, restrictions on individual liberty and the identification and care of vulnerable populations to the obligations owed to health-care workers, among many other issues. In these highly uncertain times, governments and the public alike crave competent and balanced ethical advice. Encouragingly, several national ethics committees were able to quickly produce recommendations. In Europe, for instance, a dedicated website listed advisories from 15 national ethics committees less than 2 months after the pandemic was declared.^29^ The committees in many low- and middle-income countries have been less visible, as reported in a web-based discussion organized by UNESCO in May 2020 and as evidenced by the lack of information on their websites.^30^ Although ascribing any particular reason for this lack of public visibility is difficult, it could be linked to a lack of capacity, resources or motivation – 40–50% of national ethics committees in our survey reported moderate to severe challenges in these areas (Fig. 3).

In general, national ethics committees’ advice on COVID-19 related to the public health response (e.g. resource allocation, contact tracing and treatment access) and not to research generated by the pandemic.^29^ Research governance and the oversight of individual research projects are not usually the purview of national ethics committees. Research conducted in the wake of the pandemic raises its own ethical considerations, not least the need for a timely ethics review and an appropriate risk–benefit analysis. These considerations are usually dealt with by national research ethic committees or institutional ethics committees. Nonetheless, in Latin America, a network of national ethics committees was convened by UNESCO and developed an ethics advisory for biomedical research.^31^

### Challenges for national ethics committees

Our survey revealed that many national ethics committees suffer a general lack of resources and face challenges in several domains (Fig. 3). Here we discuss the influence of these problems on key issues such as sustainability, effectiveness, impact, accountability and independence and propose solutions.

#### S*ustainability*

Whether or not a national ethics committee is relevant, active and productive in the long run is ultimately decisive for its success or failure. It is critical that committees: (i) have ethical expertise; (ii) keep up to date on scientific and technological progress; (iii) are connected internationally; and (iv) are able to react to new developments in a timely manner, be it through expert consultations, literature reviews or international and regional networking and collaboration.

Experience has shown and our survey confirms, however, that many national ethics committees struggle to fulfil their roles. Evaluations of UNESCO’s Assisting Bioethics Committees programme have found that established committees frequently do not have sustained backing from governments, independence or a pluralist make-up.^32^^–^^34^ Their struggles can largely be attributed to inadequate funding: committees can only function properly if they have the means to pay, for example, for meetings, administration, travel, training and premises. It appears that, although many governments have agreed (perhaps under international pressure) to establish national ethics committees, their commitment to providing adequate resources for a well functioning and vibrant committee is less than optimum. In our opinion, and looking at European models, national ethics committees that have been established through a legal or juridical process are more likely to be sustainable over the long term because a juridical process implies at least some support from the public. Therefore, to be sustainable, committees should ideally be established through an inclusive mechanism, involving broad public consultation and appropriate legal safeguards, and be provided with adequate resources.

#### Effectiveness

If the purpose of national ethics committees is to advise, then it is worrying that almost a quarter (21/87) of committees surveyed did not make any ethics recommendations to their governments in 2017. Moreover, the median number of reports, opinions or recommendations issued was only two per committee (Table 1). Ultimately the benchmark of a successful national ethics committee should be: (i) its output (i.e. recommendations and advisories); and (ii) its effectiveness, namely, its influence on policy-making and legislation and its contribution to public bioethics education. Therefore, national ethics committees must be set up to ensure that law-makers seriously consider their advice and committees should strive to be visible through proactively providing opinions and recommendations. Although measuring output in terms of recommendations and other publications might be straightforward, gauging effectiveness is more difficult and requires committees to individually reflect on their own performance.

#### Political environment

We are aware that a national ethics committee’s effectiveness does not only depend on adequate resources: the policy environment and the wider context in which a committee is asked to provide advice are critical. For instance, how much of a country’s policy-making is driven by values such as integrity, competence, dignity, respect for human rights and transparency? Although the survey did not attempt to examine these factors, we believe that, in a weak policy environment, national ethics committees have an uphill task and may be more effective if they increased their focus on public engagement and strove to form durable links between science, society and policy-makers rather than attempting to advise policy-makers who may have little or no regard for such advice.

#### Accountability, transparency and public engagement

In the past, national ethics committees have been heavily criticized for “muddling through” without providing ethical justifications for their recommendations.^35^ Committees should account for their recommendations, report how they came to their conclusions and describe the arguments underpinning their recommendations. By making public details of their composition, decision-making processes, recommendations and funding, national ethics committees can increase transparency, foster relationships with the public and fulfil the goal of informing the public about ethical matters. These tasks should be a central part of a committee’s mission and must not be sidelined. Committees should draw attention to topics that are highly relevant to the general public, not just to professional law-makers. Regrettably, our survey revealed that many committees did not issue press releases, nor did they engage with the broadcast media. Likewise, social media use was very low. Therefore, national ethics committees should invest in better public engagement and strive to work more effectively with the media. Creating and disseminating educational material on bioethics for a variety of audiences is another possibility.^36^ Being visible to, and appreciated by, the general public will likely lead to more sustained attention from policy-makers.

#### Independence

National ethics committees should not be politically controlled or sanctioned for their decisions: they must remain independent advisory bodies. In our opinion, independence from government, religious institutions, funding bodies, industry and political groups, among others, is key to the success and credibility of committees as they are generally expected to provide advice on highly controversial and political matters. One cannot expect national ethics committees and their members to provide rigorous and independent ethical scrutiny if they fear any form of retaliation. However, committees should not distance themselves from governments: links to government can provide privileged access to information, raise the committee’s profile and be politically useful.^3^ Committees must, therefore, walk a fine line between proximity to policy-makers and intellectual and political independence. However, it is not rare for governments to establish a national ethics committee, appoint its members and chair, provide funding and premises, and exercise some form of control. Only 40.2% (35/87) of committees surveyed expressed no concerns about independence. Thus, determining how independence is understood by different national ethics committees and how it can be improved is important. It may even be possible to arrive at a universal agreement on what it means to be an independent national ethics committee.

## Conclusion

Even though the term national ethics committee subsumes distinct entities around the globe, each with their own issues, weaknesses and strong points, there are common characteristics and challenges. Given the vast scale and societal significance of the issues dealt with by these committees, global and national efforts are required to raise awareness that national ethics committees should be strengthened to enable them to provide high-quality advice. If national ethics committees and their advice are to matter, they must be legally mandated, independent, diverse in membership, transparent and sufficiently funded to be effective and visible. Governments might see ethics committees as additional obstacles that delay the implementation of policy but that is perhaps preferable to the risk that policy will be derailed after its implementation because of the failure to take societal values and preferences into account. Especially in times of great uncertainty when drastic measures must be taken, such as during the COVID-19 pandemic, governments would be well advised to base their actions not only on technical considerations but also on the ethical guidance provided by a national ethics committee. Involving national ethics committees will increase compliance with public health measures by engendering credibility and public trust – assets most needed in such times.
